# Assessment of Intuitive Eating and Mindful Eating among Higher Education Students: A Systematic Review

**DOI:** 10.3390/healthcare12050572

**Published:** 2024-02-29

**Authors:** Fabiane Rezende, Bruno M. P. M. Oliveira, Rui Poínhos

**Affiliations:** 1 Faculty of Nutrition and Food Sciences, University of Porto (FCNAUP), Rua do Campo Alegre 823, 4150-180 Porto, Portugal; fabianerezende@fcna.up.pt; 2 Laboratory of Artificial Intelligence and Decision Support, Institute for Systems and Computer Engineering, Technology and Science (LIIAD, INESC-TEC), 4200-465 Porto, Portugal; bmpmo@fcna.up.pt

**Keywords:** higher education students, intuitive eating, mindful eating, eating behaviors

## Abstract

Background: The role of mindful eating (ME) and intuitive eating (IE) in improving eating behavior, diet quality, and health is an area of increasing interest. Objective: The objective of this review was to identify the instruments used to assess ME and IE among higher education students and outcomes related to these dimensions. Methods: This review was carried out according to the PRISMA statement, through systematic searches in PubMed, Web of Science, PsycInfo, and Scopus. The inclusion criteria selected for higher education students, levels of ME and/or IE reported, and observational and clinical studies. The exclusion criteria selected against reviews, qualitative studies, and case studies. Quality was assessed using the Academy of Nutrition and Dietetics Quality Criteria Checklist. Results: A total of 516 initial records were identified, from which 75 were included. Cross-sectional studies were the most common research design (86.7%). Most studies were conducted with samples that were predominantly female (90.7%), White (76.0%), aged 18 to 22 years (88.4%), with BMI < 25 kg/m^2^ (83.0%), and in the United States (61.3%). The Intuitive Eating Scale (IES), the Mindful Eating Questionnaire (MEQ), and their different versions were the most used instruments. The outcomes most studies included were eating behavior and disorders (77.3%), anthropometric assessments (47.8%), mental health (42.0%), and body image (40.6%). Regarding the quality of studies, 34.7% of studies were assigned a positive, 1.3% a negative, and 64.0% a neutral rate. Conclusions: IES and MEQ were the most used instruments. RCT and cohort studies are scarce, and future research with a higher level of quality is needed, especially on the topics of food consumption, diet quality, and biochemical markers.

## 1. Introduction

In recent years, mindful eating (ME) and intuitive eating (IE) as psychological function approaches have not only received considerable research interest but have also been frequently applied in clinical contexts to address problematic eating behaviors and the challenges many face in controlling their food intake [1,2,3]. The practices of ME and IE have also been used to influence energy intake or diet quality, but the evidence is still insufficient to draw strong conclusions about their effects on food consumption [4] and on weight management [5,6].

ME arose in the context of the investigation of mindfulness-based interventions initiated by Jon Kabat-Zinn (1982) [7] in the late 1970s. It corresponds to the enjoyment of food utilizing all the senses, without judgment, listening to internal cues of the body (i.e., hunger and satiety) to avoid overconsumption, and utilizing external cues (reducing portion sizes and distractions while eating, and eating slowly) to assist in achieving awareness [8]. The first studies on ME began in the 1990s, in the context of binge eating [2], and since then, different measurement scales of ME scores have been developed [9].

IE is a style of eating that focuses on eating motivated by physical reasons, being characterized by eating based on physiological hunger and satiety cues rather than situational and emotional cues, and it is associated with psychological well-being [10]. The first IE measurement scales appeared in the 2000s, and since 2006, Tylka et al. [10,11] have been deepening the study of its psychometric properties and improving the Intuitive Eating Scale.

Entering university can be a moment in life marked by great social pressure, with situations and challenges that increase the levels of stress, anxiety, and depressive symptoms [12], contributing to an increased risk of dysregulation of eating and worsening of eating behaviors [13] and body image perception, leaving university students more vulnerable to eating disorders [14]. In this context, ME and IE are useful approaches to promote improvements in eating and mental health by helping students to focus on their own cues of hunger and satiety, rather than following fashion trends or giving in to social pressure [15,16].

Most recent studies confirm that eating disorders are highly prevalent worldwide, especially in women [17], and the burden of eating disorders peaks at 25 to 29 years for females and 30 to 34 years for males [18]. In addition, authors point out that the pandemic has brought new challenges and obstacles for those who have a problematic relationship with food [19]. During the pandemic, the incidence of a first diagnosis of an eating disorder increased with an overall excess of 15.3% compared with the previous year and was greater in adolescents aged between 10 and 19 years old [20].

In view of this, there is a growing interest in the study of approaches focused on eating behavior and the dimensions of eating behavior, especially ME and IE. This systematic review examines the evidence from primary studies that evaluated ME and IE with the aims (1) to describe the scales used to measure ME and IE in college students and (2) to identify the outcomes related to ME and IE.

## 2. Materials and Methods

This systematic review was performed in accordance with the PRISMA (Preferred Reporting Items for Systematic Reviews and Meta-Analyses) guidelines [21]. The protocol of this review was registered in PROSPERO (registration number CRD42022358570). This review investigates the following question: Which instruments have been used to measure mindful eating and intuitive eating among higher education students?

### 2.1. Search Strategy

Searches for peer-reviewed journal articles were performed in Scopus, Web of Science, PsycInfo, and MEDLINE/PubMed. There were no restrictions on language or year of publication. The databases were searched using key phrases and Boolean operators that were established based on the PICO (Problem, Intervention, Comparison, and Outcome) criteria (Table 1). Web of Science, Scopus, PsycInfo, and Pubmed were searched up to 3 November 2023. The literature search was performed using the following terms without restrictions (“intuitive eating” OR “mindful eating” OR (mindfulness AND (eating OR food OR diet*))) AND (“higher education students” OR “university students” OR “college students”). The reference lists of selected studies were hand-searched, and additional references were included if relevant and if not retrieved by the initial database searches.

### 2.2. Inclusion and Exclusion Criteria

The following inclusion criteria were used: studies with higher education students of one or both sexes; studies that evaluated the levels of ME and/or IE; observational (cohort, cross-sectional, and case–control studies) and clinical studies. Systematic reviews, meta-analyses, literature reviews, qualitative studies, and case studies were excluded. All studies presenting original empirical results and meeting the other eligibility criteria were included in the review. 

### 2.3. Study Selection

**Figure 1:**
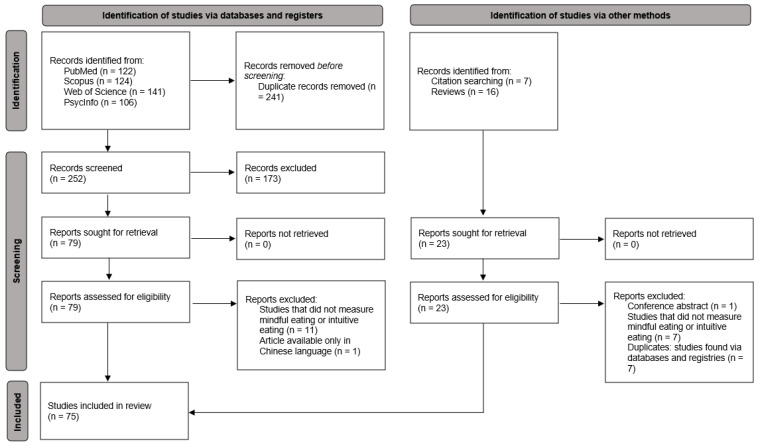
PRISMA flow diagram illustrating the identification of studies.

The study selection process was performed independently by two reviewers (F.R. and R.P.) using EndNote20 reference management software. Duplicate studies were removed. Title and abstract screening, followed by full-text screening, was performed against the eligibility criteria. The two review authors independently screened the titles and abstracts of the articles identified in the searches. Full texts were obtained for all studies considered eligible for inclusion from this process or for which eligibility was unclear. The two review authors independently decided on which studies to include or exclude. Any disagreements were resolved by discussion, and if consensus was not reached, another review author (B.O.) not involved in the search process was consulted and a decision made. Reasons for exclusion were noted by each author, discussed, decided upon as a group, and recorded in the PRISMA flow diagram (Figure 1).

### 2.4. Data Extraction

Two of the review authors independently extracted data using a standard data extraction form developed by the review authors for the purpose of this review according to the PICO model. 

The following data from each included study were extracted: (1) general: authors, year of publication, country; (2) study design; (3) sample characteristics: size, sex, age, ethnicity, body mass index (BMI); (4) ME and IE measurement scales; (5) outcomes associated to ME and IE.

### 2.5. Quality Assessment

Quality was assessed using the Academy of Nutrition and Dietetics Quality Criteria Checklist: Primary Research tool [22]. This tool consists of a questionnaire to evaluate the validity of 10 study-related items: (1) research question, (2) selection of participants, (3) comparability of study groups, (4) handling of withdrawals, (5) blinding, (6) adequate intervention detail, (7) outcome reliability, (8) appropriateness of statistical analysis, (9) conclusion accuracy, and (10) bias from funding or sponsorship. Studies were assigned a positive rating (if positive for items 2, 3, 6, and 7 and for at least one additional item), negative rating (if negative for 6 or more items), or neutral rating (if items 2, 3, 6 and 7 indicated that the study was not exceptionally strong).

### 2.6. Data Synthesis

Study data were explored according to the PICO strategy, and for each study included in this review, the following were described: the sociodemographic profile (sex, age, ethnicity) and BMI (mean, SD, and BMI categories) of higher education students. In addition, factors associated with ME and IE were described, divided into the following categories: eating behavior(s) and eating disorders; food intake and diet quality; BMI and other anthropometric or body composition assessments; body image; mindfulness; self-compassion; physical activity; quality of life and mental health; and biochemical markers.

## 3. Results

### 3.1. Study Selection

A total of 387 studies resulted from searches of the following: PubMed (*n* = 122), Scopus (*n* = 124), Web of Science (*n* = 141), PsycInfo (*n* = 106), and records identified in other sources (*n* = 23). After the removal of duplicates, 275 studies were examined for title and abstract screening; 102 study reports remained for full-text screening; and 75 studies met the final criteria for inclusion in the review. An overview of the study selection process is shown in Figure 1. The extraction of the main information from the studies is presented in chronological order (Table 2). The proportions calculated during data extraction from the studies were obtained considering the total number of studies included in the review (*n* = 75).

### 3.2. Study Design and Quality

**Figure 2:**
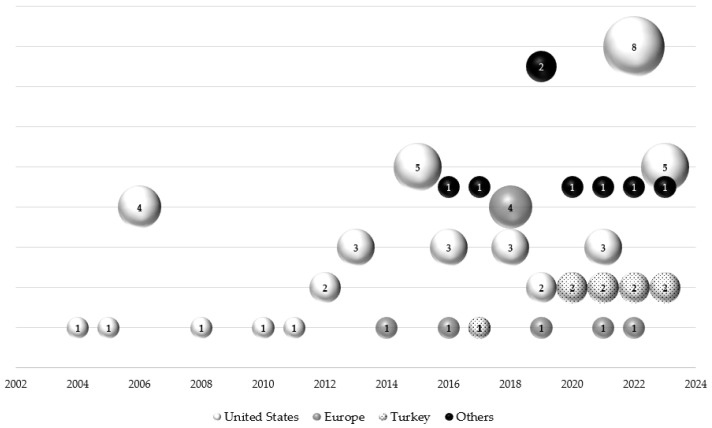
Countries and years of publication of the studies included in this systematic review.

The publication of the studies occurred predominantly in the last decade (2014 to 2023) (*n* = 61, 81.3%) (Figure 2). Of the 75 studies included in the final review, 65 (86.7%) were cross-sectional, 6 (8%) were randomized clinical trials (RCT), 3 (4%) were quasi-experimental, and 1 (1.3%) was a randomized quantitative crossover study. The duration of RCT interventions ranged from 1 to 16 weeks, including follow-up time after the intervention. 

Upon evaluation per the Academy of Nutrition and Dietetics Quality Criteria Checklist, 34.7% (*n* = 26) studies were assigned a positive rating, 1.3% (*n* = 1) a negative rating, and 64% (*n* = 48) a neutral rating. 

### 3.3. Participant Characteristics

Most of the studies were carried out in the United States (*n* = 46, 61.3%), followed by Europe (*n* = 12, 16%), Turkey (*n* = 9, 12%), and other countries (*n* = 8, 10.7%) (Figure 2). 

The numbers of participants in individual studies ranged from 14 to 2133; from the 75 studies, 68 (90.7%) had samples with a higher proportion of women, and 20 (26.6%) were conducted exclusively with female participants. In most studies (*n* = 61, 88.4%), the age of the participants was between 18 and 22 years old (based on the mean, median, or frequencies). Among the studies that assessed BMI (53 out of 75; 70.6%), in most of them (*n* = 44, 83.0%), the mean BMI was below 25 kg/m^2^, and the BMI ranged between 13.3 and 59.06. In none of the studies was the average greater than 30 kg/m^2^. Among the studies that described ethnicity (50 of 75; 66.6%), in most of them (*n* = 38, 76%), more than half of the sample of participants was White.

### 3.4. ME and IE Measurement

Among the studies that measured IE (*n* = 51), the IES proposed by Tylka (2006) [10] and its different versions were the more frequently used scales (*n* = 46, 90.2%). Among the studies that measured ME (*n* = 27), MEQ [34] and its different versions were used in approximately two-thirds (*n* = 17, 62.9%) (Table 3). Of the total collection of studies, four used IES-2 subscales and one used an MEQ subscale (Table 2). 

### 3.5. Outcomes

**Figure 3:**
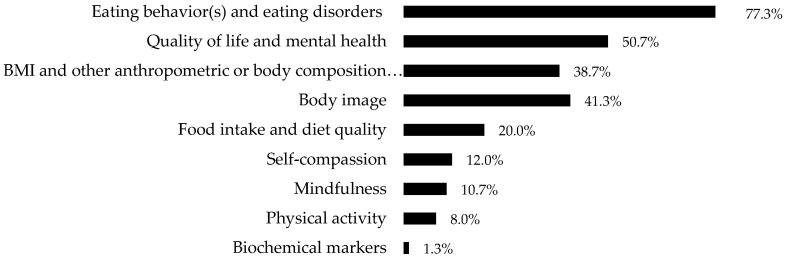
Outcomes evaluated in the studies included in this systematic review.

The outcomes were grouped by category, and the frequencies with which they were evaluated in the studies were as follows: eating behavior(s) and eating disorders (*n* = 58, 77.3%); quality of life and mental health (*n* = 38, 50.7%); BMI and other anthropometric or body composition assessments (*n* = 29, 38.7%); body image (*n* = 31, 41.3%); food intake and diet quality (*n* = 15, 20%); self-compassion (*n* = 9, 12.0%); mindfulness (*n* = 8, 10.6%); physical activity (*n* = 6, 8.0%); and biochemical markers (*n* = 1, 1.3%) (Figure 3).

## 4. Discussion

In this systematic review, the objective was to analyze the state of the art of research on ME and IE among higher education students. It was found that the studies predominantly involved young, female, and White participants, with average BMI values in the normal weight category. Furthermore, it was observed that although research on ME and IE has been ongoing for over two decades, the increase in the number of publications has been more significant in the last 10 years.

ME and IE are two important concepts in the field of nutrition, health, and eating behavior that can be particularly relevant for higher education students who are in a transitional phase from adolescence to adulthood, with significant demands for adaptation in their routines, including eating habits, new responsibilities, new relationships, and academic activities that require time, concentration, and performance evaluation [99,100]. These factors can influence eating behavior and patterns and require greater attentional and emotional regulation [101,102].

Although the fundamental principles of ME and IE are universal and can be applied in any culture or context, the cultural context can affect how people perceive and practice ME and IE. This systematic review found that most studies focused on North America, especially the United States, which has been a major country in ME and IE research. However, these concepts have also been studied in other countries, including Turkey, Canada, United Kingdom, Japan, and others. In countries like Japan, researchers have proposed ME scales with expanded dimensions to encompass aspects such as health promotion and sustainability [65]. Given the cultural and social differences, it is important for researchers to be aware of the necessary adaptations for different cultural contexts, in terms of both measurement scales and intervention protocols.

Based on the studies analyzed in this review, it was found that the scales most frequently used for measuring IE were the Intuitive Eating Scale [10] and its different versions, and those for measuring ME were the Mindful Eating Questionnaire [34] and its different versions. Studies on the psychometric properties of the IE scales have been more consistent and systematic for the IES [10,11], which is based on four constructs: Body–Food Congruence, Eating for Physical Reasons and not Emotional Reasons, Reliance on Hunger and Satiety Cues, and Unconditional Permission to Eat. On the other hand, the scales for ME have important differences in their conceptual bases and psychometric properties. One factor that has likely contributed to this is the lack of a clear definition of ME.

Mantzios [9] points out that the lack of a clear definition of ME has resulted in variations in its description in the academic and clinical literature, as well as different psychometric tools, which interfere with comparisons of evidence between studies and the quality of evidence produced from clinical interventions. In addition to the discussions regarding semantics, there is a central problem in the definition of mindful eating: the attention to and perception of hunger and satiety during the meal results in a conflicting feedback loop for the ability to maintain a posture without judgment, as it ends up interfering, for example, when making decisions about eating [9]. MEQ [34] and MES [36] are the scales most frequently used in studies, and although both are useful for measuring attention specifically focused on eating behavior, they have important issues to be discussed, especially regarding how they were developed and the constructs they comprise. The MEQ measures five constructs: disinhibition, awareness, external influences, emotional response, and distraction [34], while the MES measures six constructs: acceptance, awareness, non-reactivity, acting with awareness, routine, and non-structured eating [36]. 

Although the MEQ [36] was the first instrument proposed to measure ME, it was conceived based on items and constructs from various existing scales for assessing eating behavior and mindfulness, and there is the possibility of overlap between the constructs due to the selected items used to compose the scale. On the other hand, the MES [36] was proposed in a manner more consistent with the standard definitions of mindfulness, and its validity was assessed based on outcomes to which mindfulness-based interventions apply. However, it has the limitation of being conceived in a study involving a small (*n* = 127) and predominantly female (77.2%) sample of students.

Therefore, researchers must be aware of the limitations when choosing a scale to measure and interpret the results of research on ME and IE and, whenever possible, culturally adapt and test the reliability and validity of these scales before applying them to the target population. Low Cronbach’s alpha values condition the reliability of the data. Therefore, researchers should prefer scales with high Cronbach values and ideally measure the alpha value each time the test is administered. Despite the limitations pointed out in this review, the currently available scales have allowed the measurement of ME and IE and the exploration of these concepts in different aspects of physical and mental health in the university population.

Another aspect identified in this review was the diversity of outcomes studied regarding ME and IE. The most frequently investigated outcomes were eating behavior and eating disorders; anthropometric measures, especially BMI; mental health; and body image. Studies including biochemical markers were scarce, possibly due to them being more expensive and complex to conduct.

Regarding the effects of ME and IE approaches in the university population, the evidence is limited due to the scarcity of clinical trials. It is still uncertain whether students can benefit from the effects of these approaches based on results from other studies conducted with the general population [103]. In terms of psychological aspects, IE has been inversely associated with multiple indices of pathological eating, body image disturbances, and psychopathology and positively associated with positive psychological constructs such as positive body image, self-esteem, and well-being [15,104]. According to a meta-analysis of clinical trials, mindfulness-based interventions have resulted in improvements in mindfulness scores and binge eating symptoms [105,106]. Additionally, a meta-analysis of clinical trials found a significant weight loss effect of ME/IE strategies compared to no-intervention controls. However, these effects were not different from those observed for conventional diet programs [6]. Regarding the influence of ME and IE on energy intake or diet quality, a systematic review conducted by Grider et al. [4] pointed out that the evidence is still too limited to draw strong conclusions, and the authors suggest high-quality study designs for future research.

IE and ME are not centered on body weight and weight loss, and in the scientific literature, it is not fully understood whether and how these approaches may affect weight development. Some studies have suggested that IE is inversely associated with maladaptive eating behaviors, such as restrained, emotional, and external eating [107], and that ME and IE could be a practical approach to weight control; however, the effects that are observed when ME and IE are compared to non-intervention controls are no longer observed when ME and IE interventions are compared to conventional diet programs [6].

So far, we are not aware of any studies that have compared the effects of ME and IE on health, and it is not possible to say whether there is any type of advantage of one approach over the other. Both include the process of being mindful about eating without judgment, connecting with bodily sensations and sensory experiences with food, noticing hunger and satiety, and making conscious food choices. However, Kerin et al. [108] studied the associations between the IES and MES subscales, and they showed that some associations were small or nonsignificant, suggesting that some ME and IE components have more in common than others. For example, acceptance (a subscale of the MES) showed the greatest and most consistent overlap with all three subscales of IE and with Unconditional Permission to Eat, while Eating for Physical Reasons and not Emotional Reasons showed overlap with present eating, acceptance, and acting with awareness. It is necessary to better investigate the interfaces and differences between ME and IE and to identify the extent to which these approaches produce similar or distinct effects on eating behavior and health.

Future cohort and RCT studies with university students are needed to measure the effectiveness of ME and IE interventions in promoting healthy eating and preventing and/or treating obesity and chronic diseases in this population. Considering that ME and IE can influence attention regulation, emotion regulation, and executive function [109,110], future studies could contribute to better clarify the effects of these approaches on the mental health and academic performance of higher education students.

This review contributes to clarifying the state of the art regarding ME and IE in the university population and provides important insights into the measurement scales and existing gaps in the research with higher education students in order to support researchers. A limitation of this review is that despite extensive research in various databases, there is always a possibility that some studies may have been missed.

## 5. Conclusions

Although ME and IE have received increased attention in recent years, there are still significant gaps in the scientific knowledge on the subject. It can be considered that the scientific evidence on ME and IE in higher education students is still limited, especially due to most studies being cross-sectional in nature, conducted with small sample sizes, and lacking appropriate control groups in clinical trials and longitudinal study data. Most studies are cross-sectional, of short duration, and with a predominance of female individuals, of normal weight, residing in the USA and Europe. IES-2 and MEQ were the instruments most frequently used, and the measurement of ME and IE occurred predominantly in studies related to eating behavior and psychological features. Clearly, it is important that further research better assess the effects of ME and IE on diet quality, overweight/obesity management, and cardiometabolic markers, especially cohort studies and RCT.

## Figures and Tables

**Table 1:** PICO criteria for inclusion of studies.

Parameter	Inclusion Criteria
Population	Higher education students of both sexes
Intervention (or Exposition)	Assessment of mindful eating and/or intuitive eating
Comparison	Not applicable
Outcome	Scales used to measure ME and IE and associated outcomes.

**Table 2:** Study participant characteristics for included studies measuring mindful eating and intuitive eating in higher education students.

Reference	Country	Design	Participant Characteristics	Sample Size and Groups	Intervention	ME or IE Measurement	Outcome Categories *
Hawks et al. (2004) [23]	U.S.A.	CS	Age: 20.6 (3.4); 87.7% White, 6.9% Hispanic, 5.4% others	Total: *n* = 391 females F: *n* = 163 (41.6%) M: *n* = 228 (58.4%)	NA	30-item IES [23]	1
Hawks et al. (2005) [24]	U.S.A.	CS	Age: 18 to 22	Total: *n* = 32 - High IES Scorers: *n* = 15 (46.9%) - Low IES Scorers: *n* = 17 (53.1%)	NA	21-item IES [10]	3, 7, 9
Avalos and Tylka (2006) [25]	U.S.A.	CS study 1	Age: 20.24 (5.17) [17 to 55]; 82.2% European American, 5.0% African American, 3.9% Asian American, 0.6% Native American, 8.3% others	Total: *n* = 181 females	NA	21-item IES [10]	4, 8
	U.S.A.	CS study 2	Age: 19.92 (4.60) [17 to 50], 77.6% European American, 9.1% African American, 5.0% Asian American, 2.4% Latina, 5.7% others	Total: *n* = 417 females	NA	21-item IES [10]	4, 8
Smith and Hawks (2006) [26]	U.S.A.	CS	Age: almost half were 18 to 20 y, ~98% were 18 to 26 y; nearly 90% White, 4.1% Hispanic, 2.4% Asian, 1.8% American Indian, <1% African Americans and Native Hawaiians	Total: *n* = 343 F: *n* = 136 (39.7%) M: *n* = 207 (59.8%)	NA	27-item IES [23]	1, 2
Tylka (2006) [10]	U.S.A.	CS study 1	Age: 20.85 (6.21) [17 to 61]; 87.7% White American, 3.8% Asian American, 3.1% African American, 2.8% Native American, 0.5% Latina, 3.4% others	Total: *n* = 391 females	NA	21-item IES [10]	1, 4, 8
	U.S.A.	CS study 2	Age: 19.70 (4.50) [17 to 50]: 86.2% White American, 5.3% Asian American, 3.9% African American, 2.1% Latina, 2.4% others	Total: *n* = 476 females	NA	21-item IES [10]	8
	U.S.A.	CS study 3	Age: 18.92 (3.25) [17 to 55]; 75.4% White American, 13.1% African American, 4.0% Asian American, 2.0% Latina, 3.5% International, 0.5% Native American, 1.5% others	Total: *n* = 199 females	NA	21-item IES [10]	3
	U.S.A.	CS study 4	Age: 22.07 (7.38) [17 to 55]; 94.3% White American, 2.1% African American, 0.5% Latina, 0.5% Native American, 2.6% others	Total: *n* = 194 females	NA	21-item IES [10]	1
Tylka and Wilcox (2006) [27]	U.S.A.	CS study 1	Age: 18.44 (1.02) [17 to 30]; 85.9% White American, 5.3% African American, 5.0% Asian American, 2.1% Latina, 1.8% others	Total: *n* = 338 females	NA	21-item IES [10]	3, 8
	U.S.A.	CS study 2	Age: 18.72 (2.44) [17 to 55]; 81.6% White American, 8.3% African American, 4.3% Asian American, 1.8% Latina, 3.6% others	Total: *n* = 396 females	NA	21-item IES [10]	8
Hawks et al. (2008) [28]	U.S.A.	QE	Age: 22.8 (7.6) [18 to 51]; BMI: 23.4 [19.3 to 38.2]; BMI categories: 77.8% NW, 18.5% OW, 3.7% OB; 89.7% White, 10.3% others	Total: *n* = 29 females Low-dieting: *n* = 15 High-dieting: *n* = 14	Class met twice a week for 1.5 h during a 15-week semester	30-item IES [23]	1, 8
Galloway et al. (2010) [29]	U.S.A.	CS	Age: F: 18.5 (0.95), M: 18.6 (0.95); BMI: F: 24.2 (5.3), M: 25.1 (5.6); BMI categories by sex: M: 30% OW, 11% OB, F: 17% OW, 11% OB; 96% non-Hispanic White, 3% African American, 1% Asian American	Total: *n* = 98 F: *n* = 71 (72.5%) M: *n* = 27 (27.5%)	NA	21-item IES [10]	1, 3
Shouse and Nilsson (2011) [30]	U.S.A.	CS	Age: 20.8 (1.9) [18 to 24]; 52% White American, 36% African American, 4% Asian American, 4% Hispanic, 4% others	Total: *n* = 140 females	NA	21-item IES [10]	1, 8
Brown et al. (2012) [31]	U.S.A.	CS	Age: 19.2 (2.5) [18 to 35]; 66.7% White, 18.8% Asian, 10.4% Hispanic or Latina, 8.3% Black or African American, 4.2% others	Total: *n* = 48 females	NA	21-item IES [10]	1, 4
Webb and Hardin (2012) [32]	U.S.A.	CS	Age: 18.1 (0.29); BMI: 24.2 (5.37); BMI categories: 22% OW, 11.4% OB; 40.3% Black/African American; 59.7% White/European American	Time 1: *n* = 134 females Time 2: *n* = 83 females	NA	21-item IES [10]	1, 3
Moor et al. (2013) [33]	U.S.A.	CS	Age: 25.86 (9.67) [18 to 58]; BMI: 25.2 (4.3) [16.7 to 39.4]; 84.5% White, 10.7% African American, 3.6% Asian, 1.1% American Indian	Total: *n* = 90 F: *n* = 47 (56.6%) M: *n* = 36 (43.4%)	NA	28-item MEQ [34]	3, 7
Schoenefeld and Webb (2013) [35]	U.S.A.	CS	Age: 19.48 (1.46) [18 to 24]; BMI: 23.55 (5.11); 67.4% European American, 21.1% African American, 5.8% Latina, 3.2% Asian, 1.6% American Indian, 1.0% Hawaiian or other Pacific Island	Total: *n* = 322 females	NA	21-item IES [10]	4, 6, 8
Tylka and Kroon Van Diest (2013) [11]	U.S.A.	CS study 1	Age: 20.4 (5.19) [18 to 56]; 77.3% White, 13.1% African American, 4.0% Asian American, 1.3% Latina, 0.7% Native American, 2.7% others	Total: *n* = 878 F: *n* = 487 (55.5%) M: *n* = 391 (44.5%)	NA	23-item IES-2 [11]	1
	U.S.A.	CS study 2	Age: 20.45 (5.06) [18 to 53]; BMI: F: 24.02 (5.68) [15.98 to 56.25], M: 25.38 (5.48) [16.50 to 59.06]; 81.7% White, 5.5% African American, 3.5% Asian American, 1.8% Latina, 0.1% Native American, 7.3% others	Total: *n* = 1200 F: *n* = 680 (56.6%) M: *n* = 520 (43.3%)	NA	23-item IES-2 [11]	1, 4, 8
	U.S.A.	CS study 3	Age: 20.29 (4.82) [18 to 56]; 78.4% White, 5.4% African American, 4.8% Asian American, 1.0% Latina, 0.4% Native American, 6.3% others	Total: *n* = 522 F: *n* = 238 (45.6%) M: *n* = 284 (54.4%)	NA	23-item IES-2 [11]	8
Hulbert-Williams et al. (2014) [36]	U.K.	CS	Age: 25.65 (8.89); BMI: 23.59 (3.54); 85% White, 25% others	Total: *n* = 127 F: *n* = 98 (77.2%) M: *n* = 29 (22.8%)	NA	MES [36]	1, 4, 5, 8
Anderson et al. (2015) [37]	U.S.A.	CS	Age: 19.3 (1.3); BMI: 23.0 (3.8); 65.7% White, 12.4% Black, 12.4% Asian, 9.0% others	Total: *n* = 137 F: *n* = 87 (63.5%) M: *n* = 50 (36.5%)	NA	21-item IES [10]	1, 2, 3
Gast et al. (2015) [38]	U.S.A.	CS	Age: 19.58 (2.42); BMI categories: 6.5% UW, 69.0% NW, 17.5% OW, 7.0% OB; 90% White, 4% Hispanic, 3% Asian, 1.5% Native, 1% Black, 0.5% Pacific Islander	Total: *n* = 200 females	NA	27-item IES [23]	3, 4
Humphrey et al. (2015) [39]	U.S.A.	QE	Baseline characteristics by groups: - Intervention, HAES class: Age: 19 (2.0); BMI: 23 (3); 71% White - Comparison, basic nutrition class with some HAES content: Age: 19 (1.0); BMI: 24 (6); 60.6% White - Control, traditionally taught basic nutrition class: Age: 23 (6.0); BMI: 25 (6.0); 66% White	Total: *n* = 149 - Intervention: *n* = 45, F: *n* = 34 (76%) - Comparison: *n* = 66, F: *n* = 49 (74%) - Control: *n* = 46, F: *n* = 32 (68%)	Fall (2012) to spring (2013) semesters	23-item IES-2 [11]	1, 4, 8
Taylor et al. (2015) [40]	U.S.A.	CS	Age: 19.23 (1.5) [18 to 25]; BMI: 23.02 (3.69) [17.1 to 48.7]; BMI categories: 26% OW or OB; 74% non-Hispanic White, 12% Hispanic American, 14% others	Total: *n* = 150 F: *n* = 127 (85%) M: *n* = 23 (15%)	NA	28-item MEQ [34]	1, 3, 6
Tylka and Homan (2015) [41]	U.S.A.	CS	Age: 19.62 (2.87) [18 to 47]; BMI: F: 22.59 (3.36), M: 23.79 (3.40); 88.5% White American, 5.2% African American, 2.0% Asian American, 1.6% Native American, 1.2% Latina, 1.4% others	Total: *n* = 406 F: *n* = 258 (63.5%) M: *n* = 148 (36.5%)	NA	21-item IES [10]	4, 7
Anderson et al. (2015) [42]	U.S.A.	CS	Age: 19.3 (1.3) [18 to 24]; BMI: 23 (4) [13.3 to 36.0]; 65.4% White, 13.7% African American, 12.4% Asian, 8.5% others	Total: *n* = 125 F: *n* = 94 (64.4%) M: *n* = 31 (35.6%)	NA	21-item IES [10] and 28-item MEQ [34]	1, 2
Bryan (2016) [43]	U.S.A.	QE	Age: [18 to 24]; 35% African American, 29% White, 22% Latino/Hispanic, 2% Native Hawaiian or Pacific Islander, 10% others	Total: *n* = 37 F: *n* = 22 (59.5%) M: *n* = 16 (40.5%)	Nutrition course: 50 min meetings, 3 times/week for 3 months and 20 days	28-item MEQ [34]	1
Ellis et al. (2016) [44]	U.S.A. and U.K.	CS	Age: 19.75 (1.99) [16 to 25]; BMI: 23.95 (4.66); BMI categories: 1.2% UW, 68.6% NW, 21.9% OW, 8.3% OB; 96.6% White, 2.3% Black, 1.1% Asian	Total: *n* = 170 F: *n* = 121 (71.2%) M: *n* = 49 (28.8%)	NA	21-item IES [10]	1, 3
Kelly and Stephen (2016) [45]	Canada	CS	Age: 19.7 (1.93); BMI: 22.62 (3.41); 50% White, 21% East Asian, 1.6% Southeast Asian, 4.8% Black/African, 9.7% South Asian, 1.6% Middle Eastern, 1.6% West Indian/Caribbean, 1.6% Aboriginal, 8.1% unknown	Total: *n* = 92 females	NA	23-item IES-2 [11]	1, 4, 6, 8
Webb and Hardin (2016) [46]	U.S.A.	CS	Age: 19.4 (1.5) [18 to 27]; BMI: 23.5 (4.9); BMI categories: 17.9% OW and 8.8% OB; 62% White/European American, 21% Black/African American, 4% Asian or Asian American, 6% Hispanic/Latina, <1% American Indian/Alaska Native, 7% others	Total: *n* = 333 females	NA	23-item IES-2 [11]	3, 4, 6
Bas et al. (2017) [47]	Turkey	CS	Age: 21.1 (3.2) [19 to 31]; BMI: F: 22.5 (3.6) [17.1 to 29.4], M: 23.9 (3.5) [17.2 to 31.5]; BMI categories: 8.2% UW, 69% NW, 18.6% OW, 4.2% OB	Total: *n* = 377 F: *n* = 215 (57%) M: *n* = 162 (43%)	NA	23-item IES-2 [11]	1, 3, 4
Meadwos et al. (2017) [48]	U.K.	CS	Age: 18.7 (1.3) [17 to 36]; BMI: 22.0 (3.9) [14.0 to 44.5]; BMI categories: 10.2% UW, 55.6% NW, 9.9% OW, 2.7% OB, 21.6% not available; 76% White; 3% Asian—Chinese, 6% Asian—Indian, 3% Asian—Pakistani, 2% Asian—Other, 2% Black—African, 1% Black—Caribbean, 1% White/Black Caribbean, 2% White/Asian, 1% Other—Mixed, 1% Other, and 2% declined to answer.	Total: *n* = 658 F: *n* = 592 (90%) M: *n* = 59 (9%) Not answered: *n* = 7 (1%)	NA	21-item IES [10]	1, 4, 8
Bourdier et al. (2018) [49]	France	CS	Age: 21.08 (2.77) [15 to 30], BMI: 21.84 (3.56) [13.79; 43.29]	Total: *n* = 1051 F: *n* = 802 (76.3%) M: *n* = 249 (23.7%)	NA	Emotional Eating subscale of the 23-item IES-2 [11]	1, 3, 8
Loughran et al. (2018) [50]	U.S.A.	RCT	Age: 18 (70%); 90% White	Total: *n* = 146 F: *n* = 124 (85%) M: *n* = 22 (15%) Intervention: *n* = 99 Control: *n* = 47	Five weeks long, at a rate of two per week	23-item IES-2 [11]	1, 8
Mantzios and Egan (2018) [51]	U.K.	CS	Age: 24.4 (9.7), BMI: 24.7 (5.4)	Total: *n* = 152 F: *n* = 134 (88.2%) M: *n* = 18 (11.8%)	NA	MES [36]	1, 5, 6
Mantzios et al. (2018) [52]	U.K.	CS	Age: 21 (5.1); BMI: 24.8 (5.5); 72% White, 7.7% Pakistani, 6.1% Black, 6.1% mixed, 3.4% Indian, 1.5% Bangladeshi, 1.5% Chinese, 0.8% Arab	Total: *n* = 257 F: *n* = 241 (94.5%) M: *n* = 16 (5.5%)	NA	MES [36]	1, 5, 6
Mantzios et al. (2018) [53]	U.K.	CS	Age: 21.2 (5.6); BMI: 24.7 (5.5); 66.9% White European, 2.2% South Asian, 7.0% Black, 6.9% Chinese, 4.6% others, 12.4% not disclosed	Total: *n* = 546 F: *n* = 263 (48.2%) M: *n* = 283 (51.8%)	NA	MES [36]	1, 2, 5, 6
Romano et al. (2018) [54]	U.S.A.	CS	Age: 24.4 (6.1); BMI: 24.3 (5.0); 77.3% White	Total: *n* = 902 F: *n* = 613 (68%) M: *n* = 289 (32%)	NA	23-item IES-2 [11]	1
Saunders et al. (2018) [55]	U.S.A.	CS	Age: 21.35 (3.83) [18 to 53]; BMI: 24.66 (4.93); BMI categories: 2.3% UW; 60.4% NW, 25.5% OW, 11.8% OB; 37.6% Cuban, 20.7% South American, 8.2% Central American, 4.0% Dominican, 3.6% Puerto Rican, 1.8% Mexican	Total: *n* = 482 F: *n* = 371 (77%) M: *n* = 11 (23%)	NA	23-item IES-2 [11]	1, 2, 3, 4
Webb et al. (2018) [56]	U.S.A.	CS	Age: 19.4 (1.5); BMI: 23.5 (4.9); BMI categories: 26.8% OW or OB; 62% White/European American, 21% Black/African American, 4% Asian or Asian American, 6% Hispanic or Latina, <1% American Indian/Alaska Native, 7% others	Total: *n* = 333 females	NA	28-item MEQ [35]	4, 8
Barad et al. (2019) [57]	U.S.A.	CS	Age, median (P25; P75): 20 (19; 21) [18; 29]; BMI, median (P25; P75): 22.7 (20.5; 25.1)	Total: *n* = 293 F: *n* = 221 (75.4%) M: *n* = 72 (24.6%)	NA	23-item IES-2 [11]	2, 3
Craven and Fekete (2019) [58]	U.S.A.	CS	Age: 20.10 (3.10), BMI: 27.63 (6.83); 83.7% White, 7.7% Black, 4.1% Asian, 6.1% others	Total: *n* = 196	NA	23-item IES-2 [11]	1, 4
Lyzwinski et al. (2019) [59]	Australia	RCT	Total sample: Age: 20.19 [18 to 24]; BMI: 25.91 (4.74) [21 to 43] - Intervention Group (Mindfulness App): Age: 20.16; BMI: 26.09 (4.8); 77% White - Control Group (E-Behavioral Self-Monitoring Diary): Age: 20.22; BMI: 25.73 (4.75); 71% White	Total: *n* = 90 F: *n* = 60 (67%) M: *n* = 30 (23%) - Intervention Group (Mindfulness App): *n* = 45 - Control Group (E-Behavioral Self-Monitoring Diary): *n* = 45	11 weeks	28-item MEQ [34]	1, 3, 5, 7, 8
Miller et al. (2019) [60]	Canada	CS	Age: 19.7 (1.93) [17 to 25]; 50% White, 21% East Asian, 1.6% Southeast Asian, 4% Black/African, 9.7% South Asian, 1.6% Middle Eastern, 1.6% West Indian/Caribbean, 1.6% Aboriginal, 8.1% unknown	Total: *n* = 92 females	NA	23-item IES-2 [11]	1, 3, 4
Román and Urbán (2019) [61]	Hungary	CS	Age: 21.2 (2.58) [18 to 40]; BMI: 21.9 (3.2); BMI categories: 9.3% UW, 72.8% NW, 17.9% OW, 17.9% OB	Total: *n* = 323 F: *n* = 260 (80.5%) M: *n* = 54 (16.7%) Missing: *n* = 9 (2.8%)	NA	28-item MEQ [34]	1, 3, 5, 8
Burnette and Mazzeo (2020) [62]	U.S.A.	Randomized uncontrolled pilot trial	Total: Age: 20.11 (1.99); 45.1% White - Group (eight weekly 1.5 h sessions): Age: 20.20 (1.83); 45.0% White - GSH (guided self-help for IE + eight weekly 20 min phone calls with coach): Age: 20.00 (2.21); 45.2% White	Total: *n* = 71 females - Group (eight weekly 1.5 h sessions): *n* = 40 - GSH (guided self-help for IE + eight weekly 20 min phone calls with coach): *n* = 31	16 weeks: 0 (pre-test), 8 (post-test), and 16 weeks (follow-up)	23-item IES-2 [11]	1, 4, 8
Gan and Yeoh (2020) [63]	Malasya	CS	Age: 20.9 (1.4) [18 to 25]; BMI: 21.5 (3.22); 35.4% Malay, 61.9% Chinese, 2.7% Indian	Total: *n* = 333 F: *n* = 262 (78.7%) M: *n* = 71 (21.3%)	NA	23-item IES-2 [11]	1, 3, 4, 8
Giannopoulou et al. (2020) [64]	U.K.	CS	Age: 22.48 (0.34); 46.1% studied sport and exercise sciences, 24.4% pharmacy sciences, 29.4% health sciences	Total: *n* = 221 F: *n* = 186 (84.2%) M: *n* = 35 (15.8%)	NA	28-item MEQ [34]	1, 8
Kawasaki et al. (2020) [65]	Japan	CS	Age: 20.58 (1.76); BMI: 20.21 (2.124), BMI < 18.5: 18.8%	Total: *n* = 521 females	NA	20-item EMES [65]	1, 4, 5, 8
Keyte et al. (2020) [66]	U.K.	CS	Age: 20.46 (3.25), BMI: 25.00 (7.74); 59.0% White, 24.2% Asian, 16.8% others	Total: *n* = 211 F: *n* = 188 (89.1%) M: *n* = 15 (7.1%) Missing: *n* = 8 (3.8%)	NA	MEBS [67]	1, 5, 6
Köse and Çıplak (2020) [68]	Turkey	CS	Age: 21.36 (1.88) [18 to 26], F: 21.01 (1.86), M: 21.55 (1.87); BMI: F: 21.30 (2.69), M: 23.81 (2.67)	Total: *n* = 400 F: *n* = 140 (35%) M: *n* = 260 (65%)	NA	Turkish version of the MEQ [69]	3
Köse and Çıplak (2020) [70]	Turkey	CS	Age: 21.2 (1.77); BMI: 21.92 (2.99), F: 23.38 (2.64), M: 21.03 (1.62)	Total: *n* = 368 F: *n* = 116 (31.5%) M: *n* = 252 (68.5%)	NA	Turkish version of the MEQ [69]	3, 8
Wilson et al. (2020) [71]	U.S.A.	RCT	Age: 20.6 (2.9) [18 to 30]; BMI: 23.8 (3.9) [18.34; 41.74]; 23% White, 26% Asian American, 1% Hawaiian/Pacific Islander, 2% African American, 5% Hispanic, 44% others	Total: *n* = 94 females - Intervention group: *n* = 41 - Brochure control: *n* = 53	Three time points: baseline, post-treatment, and 1-month follow-up	27-item IES [23]	1, 2, 4, 8
Kawasaki et al. (2021) [72]	Japan	CS	Age, median (P25; P75): 20 (19; 21); BMI, median (P25; P75): 20.1 (18.9 to 21.2); BMI categories: lean: 19.1%; normal: 80.9%	Total: *n* = 215 females	NA	EMES [65]	1, 2, 3
Kes and Can Cicek (2021) [73]	Turkey	CS	Age: 24.6% 18 to 20 y, 75.4% 21 to 25 y; BMI categories: 80.4% UW or NW, 18% OW, 1.6% OB.	Total: *n* = 800 F: *n* = 434 (54.25%) M: *n* = 366 (45.75%)	NA	Turkish version of the MEQ [69]	2, 3, 7
Layman et al. (2021) [74]	U.S.A.	CS	Age: 19.93 (1.45); 79.2% White/European American, 20.8% others	Total: *n* = 168 F: *n* = 119 (70.8%) M: *n* = 49 (29.2%)	NA	21-item IES [10]	4
Lopez et al. (2021) [75]	U.S.A.	CS	Age: 92% 18 to 24 y, 8% 25 y or more; 35% Asian, 24% White, 23% Hispanic, 11% Black, 6% others	Total: *n* = 758 F: *n* = 335 (44%) M: *n* = 423 (55%)	NA	23-item IES-2 [11]	1, 2
Önen and Sandikçi (2021) [76]	Turkey	CS	Age: 59.4% 18 to 21 y, 31.3% 22 to 25 y, 9.3% 26 y or above; BMI categories: 14.7% UW, 70.2% NW, 15.1% OW/OB	Total: *n* = 463 F: *n* = 295 (63.7%) M: *n* = 168 (36.3%)	NA	Turkish version of the MEQ [69]	8
Rodgers et al. (2021) [77]	U.S.A.	CS	Age: 19.84 (1.93) [18 to 25]; BMI: 22.74 (3.39); 30% health-related major; 20% natural sciences; 23% business or political science; 17% engineering, computing, or data sciences; 7% humanities; remainder: undeclared	Total: *n* = 605 F: *n* = 490 (81%) M: *n* = 115 (19%)	NA	23-item IES-2 [11]	1
Román et al. (2021) [78]	Hungary	CS	Age: 22.7 (4.81)	Total: *n* = 732 F: *n* = 587 (80.2%) M: *n* = 145 (19.8%)	NA	23-item IES-2 [11] and MES [34]	1, 3, 4
Ahlich and Rancourt (2022) [79]	U.S.A.	CS	Age: 21.12 (4.88); BMI: 24.51 (5.64); 62.8% White, 13.2% Asian, 9.8% Black or African American, 1.7% Arab or Middle Eastern, 0.4% American Indian/Alaskan Native, 11.3% others	Total: *n* = 461 Cisgender females: *n* = 244 (52.9%) Cisgender males: *n* = 209 (45.3%) Non-binary or transgender: *n* = 8 (1.7%)	NA	Reliance on Hunger and Satiety Cues subscale of the 23-item IES-2 [11]	1, 8
Belon et al. (2022) [80]	U.S.A.	CS	Age: 20 (3.2) [18 to 38]; BMI: 23.8 (4.9) [16.1 to 47.2]; BMI categories: 6% UW, 67% NW, 16% OW, 11% OB; 64% White; 44% Not Hispanic, Latina, or Spanish origin; 36% Other Hispanic, Latina, or Spanish origin; 23% Other; 20% Mexican, Mexican American, or Chicana; 8% American Indian/Alaskan Native; 4% Black/African American; 4% Unavailable/Unknown; 3% Asian	Total: *n* = 352 females	NA	23-item IES-2 [11]	1, 2, 4, 8
Cebioğlu et al. (2022) [81]	Turkey	CS	Age: 21.5 (2.2) [18 to 50]; BMI: 22.5 (3.8) [15.2 to 45.7], 20.2% BMI ≥ 25	Total: *n* = 2133 F: *n* = 1214 (56.9%) M: *n* = 919 (43.1%)	NA	Turkish version of the MEQ [69]	1, 3
Chiodo et al. (2022) [16]	U.S.A. and Italy	CS	Age: 21.79 (4.75); 29.5% non-Hispanic White American, 17.1% Hispanic American, 11.1% other Americans, 30.6% Italian, 11.7% others in Italy + missing	Total: *n* = 677 F: *n* = 466 (68.8%) M: *n* = 145 (21.4%) Missing: *n* = 66 (9.8%) Italian: *n* = 244 (36%) American: *n* = 433 (64%)	NA	20-item MEQ [82]	1, 4
Katcher et al. (2022) [83]	U.S.A.	RCT	Age: 20.9 (1.9) [18 to 26]; BMI: 26.4 (6.0) [19.9 to 41.6]	Total = 14 females Treatment group: *n* = 7 Waitlist control group: *n* = 7	Intervention period: five weeks Maintenance period: five weeks	23-item IES-2 [11]	1, 4
Lovan et al. (2022) [84]	U.S.A.	RCT	Age: 19.8 (1.43) [18 to 24]; BMI categories: 3% UW, 63.6% NW, 24.2% OW, 9.1% OB; 75.8% White, 18.2% African American, 4.5% Asian, 1.5% Native American	Total: *n* = 60 F: *n* = 36 (62.1%) M: *n* = 24 (37.9%)	Two visits, one week apart	23-item IES-2 [11]	1, 2, 3, 8
Lovan et al. (2022) [85]	U.S.A.	CS	Age: 19.8 (1.4); BMI: 24.4 (4.6), BMI categories: 3.0% UW, 63.6% NW, 24.2% OW, 9.1% OB; 5.8% White, 18.2% Black or African American, 4.5% Asian, 1.5% American Indian, 74.2% Hispanic	Total: *n* = 66 F: *n* = 41 (62.1%) M: *n* = 25 (37.8%)	NA	23-item IES-2 [11]	1, 3, 4
Mackenzie et al. (2022) [86]	Australia	Randomized quantitative crossover	Age, mean (SD): 25.25 (8.2), range: 18 to 49 y; BMI, mean (SD): 24.7 (4.9)	Total: *n* = 55 F: *n* = 41 (75%) M: *n* = 14 (25%)	One week	20-item MEQ [82]	2
Romano and Heron (2022) [87]	U.S.A.	CS	Age: 22.27 (5.83); BMI: 25.83 (6.15); 37.79% African American or Black; 0.57% American Indian and Alaska Native; 5.05% Asian, Asian American, Native Hawaiian, or Pacific Islander; 41.21% European American/White; 15.40% other	Total: *n* = 1.228 F: *n* = 931 (75.81%) M: *n* = 292 (23.78%)	NA	23-item IES-2 [11]	4, 7, 8
Shaw and Cassidy (2022) [88]	North Ireland	CS	Age: 22.04 (2.72) [18 to 30]; BMI: 25.5 (4.69); BMI categories: 11.4% UW, 41.3% NW, 35.0% OW, 2.3% OB	Total: *n* = 349 F: *n* = 244 (70%) M: *n* = 105 (30%)	NA	MEBS [67]	1, 3, 6, 8
Vrabec et al. (2022) [89]	U.S.A.	CS	Age: 19.47 (1.75) [18 to 25]; 60.2% White, 21.8% Asian or Asian American, 10.5% Black or African American, 9.4% Hispanic, 1.6% American Indian or Alaskan, 6.2% others	Total: *n* = 372 F: *n* = 238 (64%) M: *n* = 134 (36%)	NA	21-item IES [10]	1, 8
Akik and Yiğit (2022) [90]	Turkey	CS	Age: 20.82 (3.83) [18 to 27]; BMI: 22.49 (3.89)	Total: *n* = 362 F: *n* = 249 (68.8%) M: *n* = 110 (30.4%) Sex as “other”: *n* = 3 (0.8%)	NA	20-item MEQ [82]	1, 8
Cetin (2023) [91]	Turkey	CS	Age by Chronotype groups: Morning: 21.34 (2.12), Intermediate: 21.01 (1.83), Evening: 21.20 (1.70); Obesity by Chronotype groups: Morning: *n* = 2 (2.3%), Intermediate: *n* = 16 (4.0%), Evening: *n* = 6 (5.3%)	Total: *n* = 507 F: *n* = 370 (61.2%) M: *n* = 235 (38.8%)	NA	Awareness and Recognition sub-scales of the Turkish version of the 15-item MEQ [84]	1, 2, 8
Fırat and Cicek (2023) [92]	Turkey	CS	Age: 20.81 (1.85) [18 to 38]	Total: *n* = 1708 F: *n* = 899 (52.6%) M: *n* = 809 (47.4%)	NA	Turkish version of the IES-2 [47]	3
Loor et al. (2023) [93]	U.S.A.	CS	Age: 24.32 (8.41) [18 to 57]; BMI: 26.28 (6.98); BMI categories: 4.9% UW, 45.1% NW, 30.4% OW, 19.6% OB; 46.2% Hispanic, 42.3% non-Hispanic White, 5.8% Asian, 2.9% Black/African American, 1.9% American Indian/Alaska Native, and 1.0% other	Total: *n* = 104 F: *n* = 91 (87.5%) M: *n* = 13 (22.5%)	NA	23-item IES-2 [11]	1, 8
Loor et al. (2023) [94]	U.S.A.	CS	Age: 24.25 (8.38); BMI: 26.20 (6.94); 46.0% Hispanic, 41.0% non-Hispanic White, 9% Asian, 4% Black/African American, 3.0% American Indian/Alaska Native, and 2.0% other	Total: *n* = 100 F: *n* = 86 (86%) M: *n* = 11 (11%) Gender variant/non-conforming: *n* = 2 (2%)	NA	23-item IES-2 [11]	1, 8
Schueler et al. (2023) [95]	U.S.A.	CS	Age: 70.9% 18 to 19 y; BMI: 24.4 (4.6); 27.8% Hispanic or Latino, 70.9% not Hispanic or Latino, 1.3% did not say	Total: *n* = 298 F: *n* = 173 (58%) M: *n* = 125 (42%)	NA	23-item IES-2 [11] and MEBS [67]	1, 2
Yang et al. (2023) [96]	China	CS	Age: 21.12 (1.48); BMI: 20.49 (2.69); 97.3% Han, 2.7% other	Total: *n* = 702 F: *n* = 319 (45.44%) M: *n* = 383 (54.56%)	NA	23-item IES-2 [11]	1, 8
Yoon et al. (2023) [97]	U.S.A.	CS	Age: 20.9 (2.6); 15.1% non-Hispanic White, 14.1% non-Hispanic Black or African American, 33.2% Hispanic, 35.0% non-Hispanic Asian, and 2.7% others	Total: *n* = 887 F: *n* = 481 (54.2%) M: *n* = 406 (45.8%)	NA	Reliance on Hunger and Satiety Cues subscale (version adapted) of the 23-item IES-2 [11]	1, 4
Yoon et al. (2023) [98]	U.S.A.	CS	Age: 20.9 (2.7); 15.7% non-Hispanic White, 13.3% non-Hispanic Black or African American, 32.7% Hispanic, 35.6% non-Hispanic Asian, and 2.7% others	Total: *n* = 828 F: *n* = 451 (54.5%) M: *n* = 377 (45.5%)	NA	Reliance on Hunger and Satiety Cues subscale (version adapted) of the 23-item IES-2 [11]	1, 4

Notes: BMI: body mass index; CS: cross-sectional; EMES: Expanded Mindful Eating Scale; F: female; HAES: Health at Every Size; IE: intuitive eating; IES: Intuitive Eating Scale; M: male; ME: mindful eating; MES: Mindful Eating Scale; MEBS: Mindful Eating Behavior Scale; NA: not applicable; NW: normal weight; OB: obese; OW: overweight; QE: quasi-experimental; RCT: randomized clinical trial; SD: standard deviation, UW: underweight. Age expressed in years and BMI in kg/m^2^. Age and BMI reported as mean (standard deviation) [minimum; maximum], except where otherwise indicated. * Outcome categories: (1) eating behavior(s) and eating disorders; (2) food intake and diet quality; (3) BMI and other anthropometric or body composition assessments; (4) body image; (5) mindfulness; (6) self-compassion; (7) physical activity; (8) quality of life and mental health; (9) biochemical markers.

**Table 3:** Scales and questionnaires used to measure mindful eating and intuitive eating.

Instruments	*n* (%) of Studies *
***Intuitive Eating***	***51 (100%)***
30-item Intuitive Eating Scale [23]	2 (3.9%)
27-item Intuitive Eating Scale [23]	3 (5.9%)
21-item Intuitive Eating Scale [10]	16 (31.4%)
Intuitive Eating Scale 2 [11]	29 (56.9%)
Turkish version of the IES-2 [47]	1 (1.9%)
***Mindful Eating***	***27 (100%)***
28-item Mindful Eating Questionnaire [34]	8 (29.7%)
Mindful Eating Scale [36]	5 (18.5%)
Turkish version of the 30-item Mindful Eating Questionnaire [69]	5 (18.5%)
20-item Mindful Eating Questionnaire [82]	3 (11.1%)
Mindful Eating Behavior Scale [67]	3 (11.1%)
Expanded Mindful Eating Scale [65]	2 (7.4%)
Turkish version of the 15-item Mindful Eating Questionnaire [84]	1 (3.7%)

* Three studies evaluated both intuitive eating and mindful eating.

## Data Availability

Not applicable.
